# Durability Assessment of Eco-Friendly Intumescent Coatings Based on Cork and Waste Glass Fillers for Naval Fire Safety

**DOI:** 10.3390/polym17121659

**Published:** 2025-06-15

**Authors:** Elpida Piperopoulos, Giuseppe Scionti, Mario Atria, Luigi Calabrese, Antonino Valenza, Edoardo Proverbio

**Affiliations:** 1 Dipartimento di Ingegneria, Università di Messina, Contrada di Dio-Sant’Agata, 98166 Messina, Italy; giuseppe.scionti@studenti.unime.it (G.S.); lcalabrese@unime.it (L.C.); eproverbio@unime.it (E.P.); 2 Colorificio Atria, Contrada Camarro Formeca, 91028 Partanna, Italy; amario@atria.it; 3 Dipartimento di Ingegneria, Università di Palermo, Viale delle Scienze, 90128 Palermo, Italy; antonino.valenza@unipa.it

**Keywords:** intumescent coating, ammonium polyphosphate, cork, recycled glass, fire resistance

## Abstract

This research assessed novel, eco-friendly intumescent coatings utilizing cork and recycled glass as sustainable alternatives to synthetic fire retardants, aiming to reduce environmental impact while maintaining robust fire performance. Coatings underwent up to 600 h of UV light exposure for durability assessment, followed by chemo-physical characterization. Fire exposure tests evaluated in-situ char formation and foaming. All functionalized coatings exhibited suitable intumescent behavior, forming protective char layers even after extensive UV aging. Microscopic analysis showed good additive integration, while FTIR spectroscopy revealed UV-induced chemical changes. Fire resistance tests confirmed the superior performance of functionalized coatings over the commercial reference. The AP-IC system demonstrated the best intumescence, achieving significantly lower maximum temperatures (e.g., 167.3 °C for AP-IC-600) and heating rates. Crucially, the sustainable RG-IC and CK-IC batches showed promising intumescent properties, even improving with UV exposure. Notably, the foamed cross-sectional area of the aged RG-IC samples doubled compared to their unaged counterparts, reaching a maximum temperature of 166.9 °C. These findings highlight the potential of eco-friendly hybrid coatings to enhance fire safety, particularly in critical sectors like naval engineering, aligning with circular economy principles and the growing demand for sustainable, high-performance materials.

## 1. Introduction

Intumescent coatings are a critical and expanding research area, especially in industrial fields like naval applications, due to their ability to passively increase fire resistance. These materials endure high temperatures (typically 200–600 °C) for extended periods compared to standard coatings [1,2,3]. Their application on steel substrates, such as in shipbuilding for elements like bulkheads and decks, is essential for enhancing structural integrity, resilience, and safety during fire events [4,5,6]. Given the significant fire risks in harsh naval environments, these coatings are relevant for preventing collapse and protecting personnel. Continuous research into intumescent coatings is essential to meet the diverse and rigorous demands of naval applications.

Intumescent coatings protect materials from fire by forming an insulating, multi-layered foamed char when exposed to heat or flames. This expansion results from gases released by the coating’s components, creating a porous carbon-rich barrier [7,8]. This layer effectively slows heat transfer, retarding fire, reducing ignition risk, and limiting fire spread [9,10,11].

In this matter, ammonium polyphosphate (APP) is a common acid source in intumescent coatings, alongside others like boric acid and diammonium phosphate [12]. Carbonizing agents typically include pentaerythritol, starch, and mannitol [13,14], while melamine, urea, expandable graphite, and dicyandiamide often serve as foaming agents [15].

The performance of intumescent coatings is affected by various parameters like char thermal conductivity, carbon additives, or swelling initiation [16]. Consequently, designing effective intumescent coatings that optimize these agents for broader applications presents a significant challenge.

In recent years, the drive towards sustainability has significantly reshaped material science, particularly concerning flame retardant design. Researchers are increasingly turning their attention to sustainable fillers as a viable means to enhance both the environmental friendliness and effectiveness of flame-retardant materials [1,17,18]. This concept is largely driven by the growing global importance of eco-friendly methods and a concerted effort to mitigate the environmental footprint of industrial products, including intumescent coatings used for fire protection.

The effective performance of intumescent coatings over their service life is paramount, as degradation due to environmental factors can compromise their fire protective capabilities. Among these factors, ultraviolet (UV) radiation from sunlight is a relevant factor for material degradation, leading to chain scission, cross-linking, and changes in surface chemistry [19,20,21]. Therefore, assessing the UV aging resistance of sustainable intumescent coatings is indispensable for understanding their reliability and predicting their service life [22,23,24].

UV aging exposure may lead to the decomposition of the components needed for intumescence or result in physical failures of the coating, such as cracking [25]. Decomposition of the polymer binder may occur, thus reducing the performance of the thermal protection of the intumescent coating. In fact, when the polymer binder decomposes, the coating matrix loses its integrity. This may lead to loss of cohesion of the intumescent ingredients, cracking, detachment of the coating, or a poor ability to form and maintain a stable and protective char layer during a fire. If the coating fails to perform its function, the substrate will reach its critical temperature more quickly, with the risk of structural failure and a significant increase in fire hazards.

Although research has increasingly focused on incorporating sustainable fillers to enhance flame retardancy and reduce environmental impact, the comprehensive evaluation of how these fillers influence the coating’s durability, especially under prolonged UV aging exposure, remains an area still requiring extensive investigation.

This study specifically addresses this knowledge gap by investigating the durability potential of sustainable intumescent coatings incorporating cork and recycled glass as eco-friendly fillers. Cork, a renewable and naturally abundant material, offers excellent insulation properties and is a compelling sustainable alternative. Similarly, recycled glass contributes to circular economy principles while potentially offering beneficial reinforcing or barrier properties within the coating matrix. Evaluating these materials not only aligns with sustainability goals but also seeks to understand their inherent resistance to environmental issues.

Our research involved subjecting these novel coatings to accelerated UV aging tests for up to 600 h of continuous exposure. This approach aimed to simulate long-term outdoor conditions and induce potential microstructural modifications or degradations. Following UV treatment, both aged and unaged samples underwent detailed chemo-physical characterization to identify changes at the molecular and morphological level. Beyond durability, fire exposure tests were conducted to confirm that the sustainable coatings, even after aging, maintained their intumescent action, char formation, and char foaming characteristics. By assessing the durability of coatings formulated with cork and recycled glass under aging conditions, this research provides insight into their long-term durability and paves the way for their widespread use in sustainable fire protection solutions.

## 2. Materials and Methods

### 2.1. Materials and Coatings Preparation

Based on the expertise of Colorificio Atria S.r.l. (Partanna, TP, Italy), the novel coatings were developed from an acrylic intumescent coating (IC) enhanced with ammonium polyphosphate (APP—supplied by Budenheim KG, Budenheim, Germany). To improve both the intumescent properties and product sustainability, two fillers were investigated: cork (Syfar Srl, Acquedolci, Messina, Italy) and recycled glass (Sarco Srl, Marsala, TP, Italy).

All coatings were deposited on ASTM A1008 carbon steel plates (dimension: 140 mm × 70 mm and thickness: 0.8 mm), achieving an average coating thickness of 76 ± 10 µm. A bare steel plate (uC) served as a reference to better evaluate the protective and intumescent action of the coatings. Sample codes were generated by combining a basic acronym (IC for Intumescent Coating) with a two-letter abbreviation for a key modifying component (AP for Ammonium Polyphosphate, RG for Recycled Glass, and CK for Cork), e.g., AP-IC stands for Ammonium Polyphosphate-Intumescent Coating. The experimental coatings (listing codes and characteristics) are detailed in Table 1.

### 2.2. UV Aging Test

To assess their long-term durability, the prepared samples underwent accelerated weathering in a Q-Lab QUV Accelerated Weathering Test apparatus (Q-LAB, Westlake, OH, USA). The UV irradiance was set to 0.89 W/m^2^. Each sample presented an exposed area of approximately 60 cm^2^ (95 mm × 63 mm) to the UV radiation. To track the progressive effects of aging, samples were systematically extracted and characterized after 300, 600, and 900 h of continuous exposure to UVA radiation.

To easily differentiate between coating batches and their respective UV exposure times, samples were given a specific code. This code includes a suffix from Table 1, which identifies the coating batch, followed by a number indicating the duration of UV aging, e.g., CK-IC-300 denotes a coating with cork particles that underwent 300 h of UV lamp aging. Likewise, RG-IC-0 refers to a coating containing recycled glass particles with no UV aging.

### 2.3. Coatings Characterization

Fourier Transform-Infrared Spectroscopy (FT-IR) was carried out using a Spectrum Two Perkin-Elmer FT-IR Spectrophotometer (Waltham, MA, USA). These infrared spectra were specifically recorded in Attenuated Total Reflectance (ATR) mode, covering the wavenumber range from 500 to 4000 cm^−1^ at a scan rate of 0.4 cm^−1^ per second. Furthermore, morphological and elemental analyses were performed using an Environmental Scanning Electron Microscope (SEM, FEI Quanta 450, Waltham, MA, USA) to visualize surface structures, complemented by Energy-Dispersive X-ray Spectroscopy (EDAX, 20 kV acceleration voltage) for elemental composition analysis.

### 2.4. Intumescent Layer Growth Test

**Figure 1:**
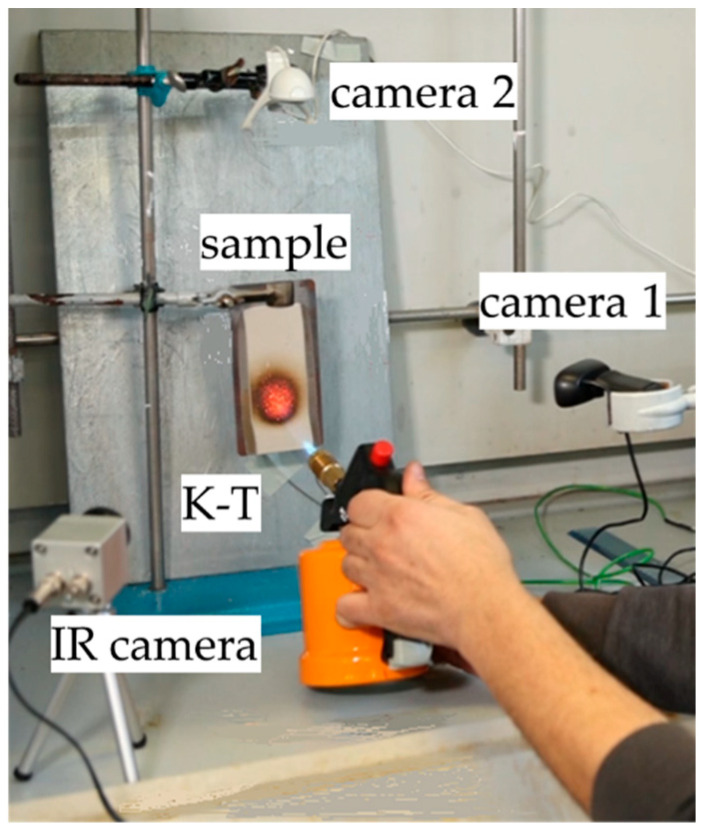
Set-up of the laboratory fire-resistance test.

A laboratory fire-resistance test was conducted to assess the direct flame reaction of the developed composite coatings. The detailed experimental setup for this test is fully presented in Figure 1. According to the test set-up, each sample was directly exposed to a gas torch flame for an approximate duration of 70 s. Throughout this period, coating modifications were progressively recorded using a system of three cameras strategically located around the sample. A thermo-camera (IR camera in Figure 1) was placed in front of the sample to monitor the temperature evolution across the sample’s top surface. Concurrently, a further camera was placed in front of the sample to assess the morphological modification of the coating during fire exposure (camera 1 in Figure 1). Finally, a lateral camera was positioned to capture the real-time evolution of the coating’s foaming profile along its cross-section during the flame exposure (camera 2 in Figure 1). Furthermore, to ascertain the coatings’ fire response capabilities and to evaluate their thermal insulation properties, the temperature evolution on the backside of the steel plate was recorded. This was achieved by attaching a K-thermocouple probe directly to the central area of the substrate’s backside. The thermocouple sensor was then connected to a data logger, which recorded temperature data at a frequency of 1 Hz throughout the test. To achieve a comprehensive assessment of the coatings’ overall fire response qualities, data acquired from the different and complementary sources was diligently examined and integrated. Moreover, the fire test was performed in triplicate for statistical validation of the results.

## 3. Results and Discussion

### 3.1. Chemo-Physical Characterization

**Figure 2:**
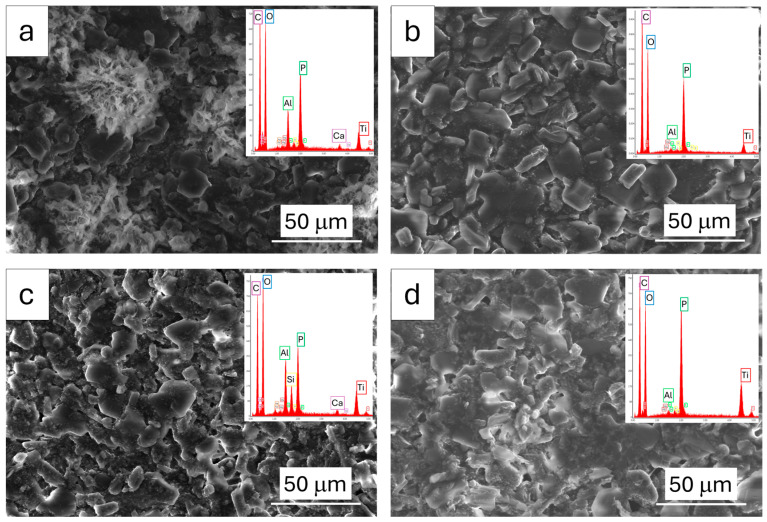
SEM images and EDX spectra (in the insets) of (**a**) IC; (**b**) AP-IC; (**c**) RG-IC; and (**d**) CK-IC coatings.

Figure 2 displays the morphology of the investigated samples.

Most coatings reveal a regular and consistent texture. However, the IC sample stands apart, exhibiting greater inhomogeneities and irregularities in its structure. Generally, the samples analyzed showcase a dense and tightly packed internal arrangement. Particles are consistently distributed throughout the matrix, appearing in both ordered patterns and more scattered configurations. This consistent distribution suggests a well-integrated composite structure for most of the coatings, with the noted exception of the IC sample’s less uniform appearance.

Furthermore, microscopic analysis provided evidence of successful material integration, as the observed surfaces were notably devoid of any discernible cracks. The absence of cracking is an indicator of the homogenous dispersion and integration of the incorporated particles within the acrylic binder. Moreover, a crack-free surface is a required aspect, as it indirectly suggests adequate material cohesion and suitable interfacial bonding between the filler particles and the polymer matrix. This inherent cohesion is important for maintaining the structural integrity of the coating, especially when subjected to the stresses of fire or environmental exposure, thereby ensuring its long-term performance and durability.

The coatings are primarily composed of pentaerythritol, melamine, and ammonium polyphosphate. EDX analysis reveals a higher amount of phosphorus (P) in the AP-IC sample, attributed to a larger percentage of ammonium polyphosphate. The carbon (C) and oxygen (O) peaks correspond to the organic matrix, along with added melamine and pentaerythritol. Additionally, peaks of magnesium (Mg), sodium (Na), aluminum (Al), titanium (Ti), calcium (Ca), and silicon (Si) are evident, indicating the presence of metal oxides and silica. Notably, titanium oxide (TiO_2_) is known to improve the thermal performance and char morphology of intumescent coatings. The higher Si peak in RG-IC is justified by the addition of silica in the matrix.

**Figure 3:**
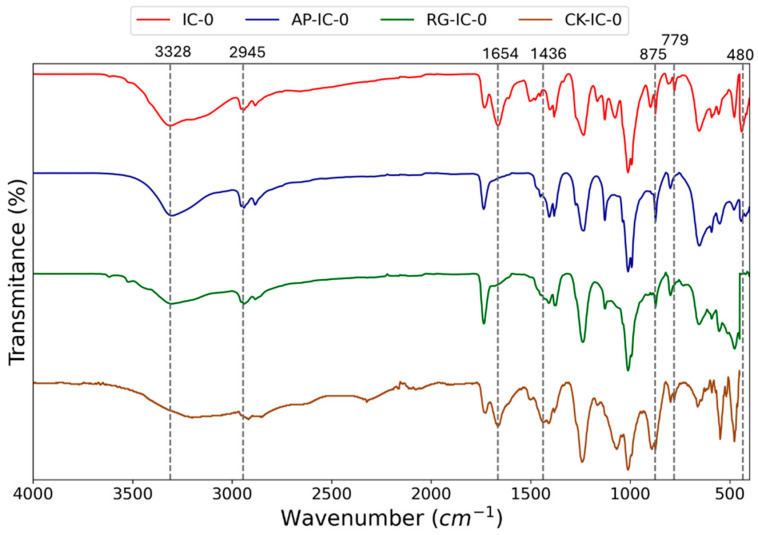
FTIR spectra of the unaged IC, AP-IC, RG-IC and CK-IC coatings.

Further insights into chemical bonding can be drawn by comparing the FTIR spectra of the coatings. Figure 3 presents these spectra specifically for the unaged coatings, providing a baseline understanding of their chemical composition before any environmental degradation.

All the samples display the typical peaks of intumescent acrylic coatings, composed of melamine, pentaerythritol, and ammonium polyphosphate. The peak at 3328 cm^−1^ indicates the pentaerythritol stretching vibration of O-H, while the peak at 2945 cm^−1^ refers to the pentaerythritol asymmetric stretching vibration of CH_3_. The peak at 1654 cm^−1^ indicates the stretching vibration of C=N, typical of the melamine FTIR spectrum [26]. The peak at 1436 cm^−1^ corresponds to C-H group vibrations in the CH_2_ chain and asymmetric angular vibrations of NH_4_ [27]. Peaks at 875 cm^−1^, 779 cm^−1^, and 480 cm^−1^ are indicative of the stretching vibrations of P-O-P in ammonium polyphosphate [26,28]. Some differences are evident between the investigated samples. Peaks at 3328 cm^−1^ and 2945 cm^−1^ appear clearer in the IC, AP-IC, and RG-IC samples, while the peak at 1654 cm^−1^ is more evident in IC and CK-IC. Peaks at higher wavenumber are present in all the samples.

**Figure 4:**
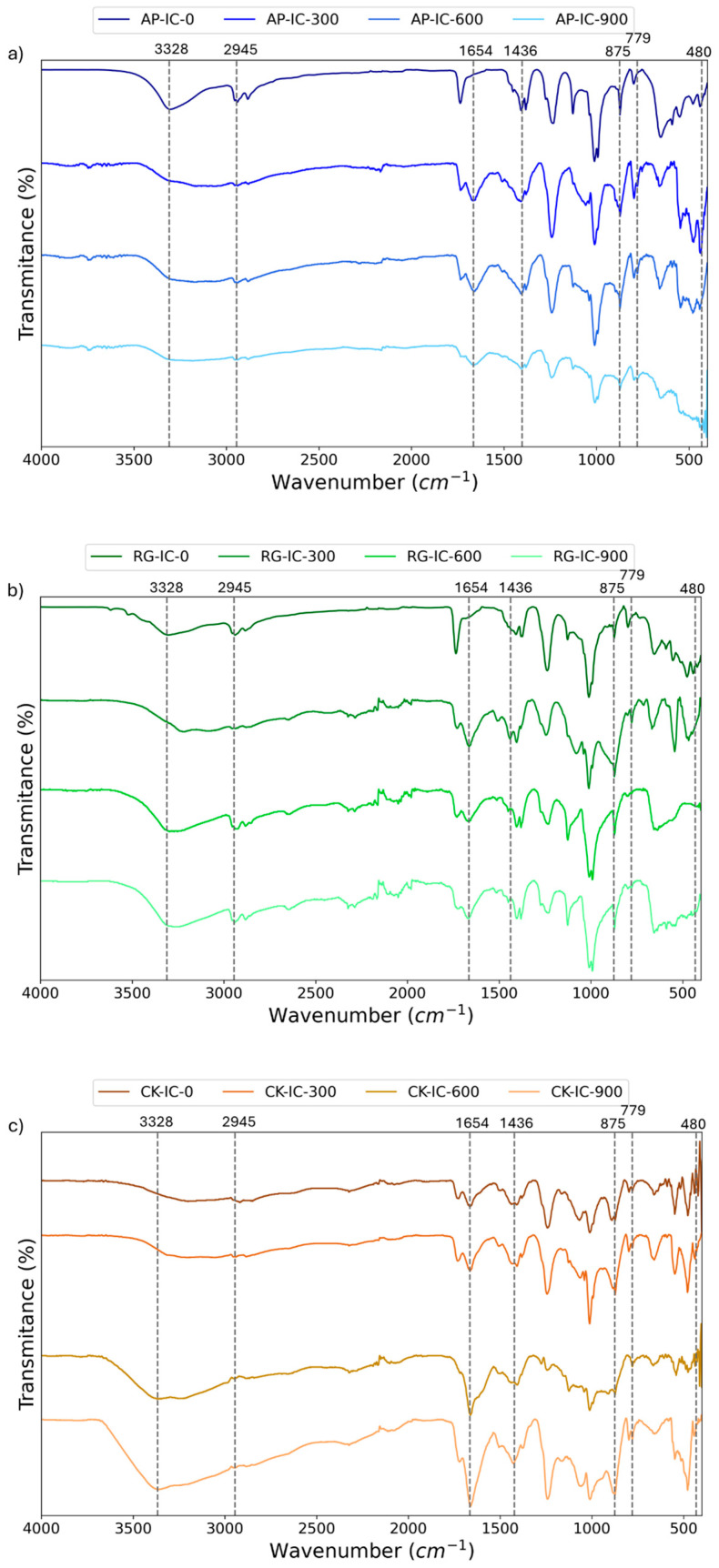
FTIR of aged (**a**) AP-IC, (**b**) CK-IC, and (**c**) RG-IC.

All prepared samples underwent FT-IR analysis to determine the structural stability of the coatings after aging. For illustrative purposes, the spectra for the AP-IC (increased APP), RG-IC (recycled glass enhanced), and CK-IC (cork powder enhanced) coatings are presented in Figure 4a, Figure 4b, and Figure 4c, respectively.

Across all coating formulations, an observable trend with increasing aging time was the intensification of the peaks at 3328 cm^−1^ and 1654 cm^−1^ in the CK-IC and RG-IC samples. This rise is attributed to the decomposition of pentaerythritol and acrylic resin, producing alcohols containing O-H and the formation of C=O groups [26], specifically acids and aldehydes, which are byproducts of the photo-oxidation of the resin and pentaerythritol. Concurrently, the peak at 1436 cm^−1^ weakened, particularly in the AP-IC sample. This weakening suggests chain scission and oxidative reactions involving the resin and ammonium polyphosphate [29], resulting in reduced containment of CH_2_ and NH_4_ groups. Furthermore, aging led to an intensification of peaks at 875 cm^−1^, 779 cm^−1^, and 480cm^−1^. These specific peaks are indicative of the stretching vibration of P-O-P in pyrophosphate [28], a compound generated through the hydrolysis of ammonium polyphosphate, providing clear evidence of chemical changes occurring due to the aging process.

### 3.2. Fire Resistance Test of the Intumescent Coatings

**Figure 5:**
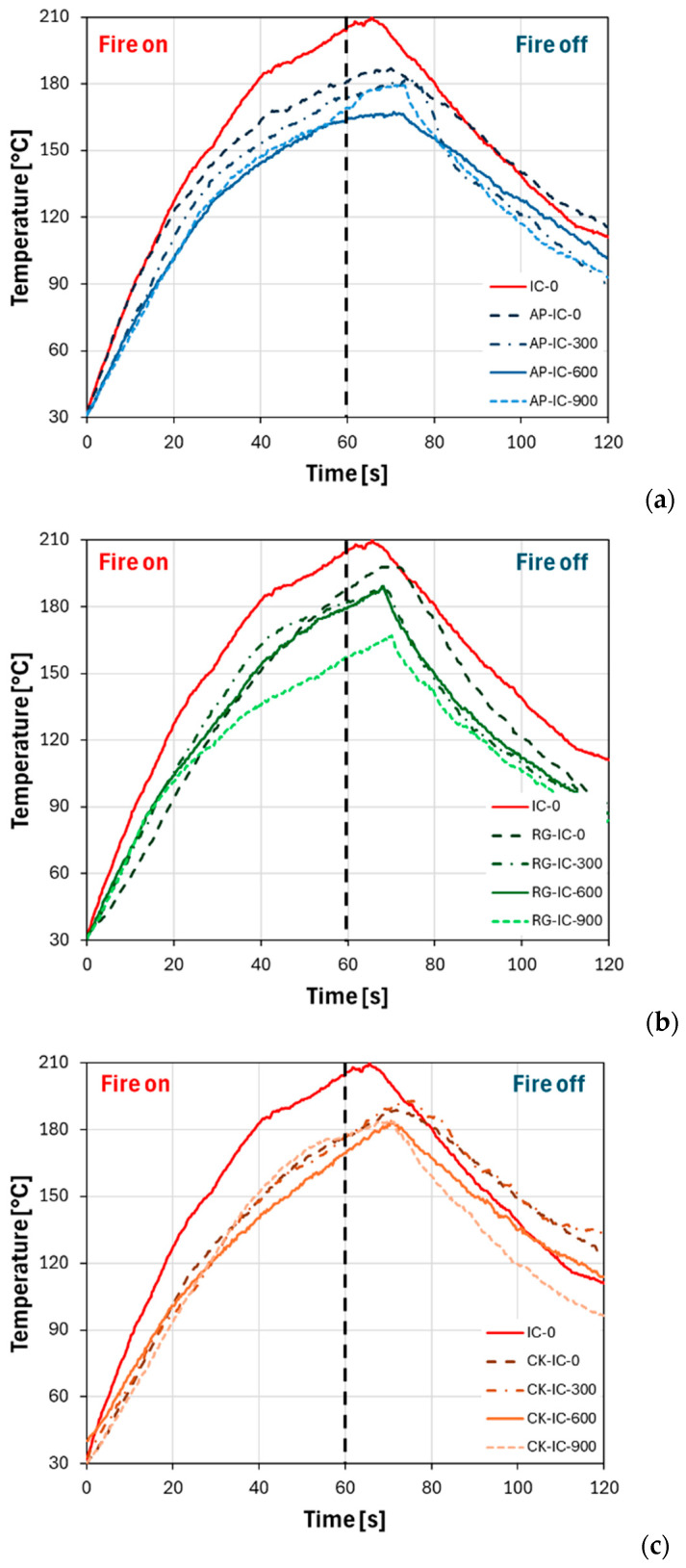
Backside temperature evolution of (**a**) AP-IC, (**b**) RG-IC, and (**c**) CK-IC coatings with increasing UV aging time. IC coating as reference.

Figure 5 illustrates the backside temperature evolution for all intumescent coatings at varying UV aging exposure times during fire resistance testing. The commercial intumescent (IC sample) coatings were also reported as a reference. To preserve the clarity and readability of Figure 5, the back-side temperature evolution of the uncoated steel sample (SS) is provided in the Supplementary Materials Section.

As expected, the uncoated steel support (SS) showed a rapid temperature increase in the first stage of the test, reaching approximately 100 °C after only 9 s of direct flame exposure. At the fire exposition’s conclusion, the SS sample reached a maximum temperature above 400 °C, confirming its minimal thermal barrier action supplied by the metal substrate. In contrast, all intumescent-coated samples displayed a qualitatively similar temperature trend, though some differences can be observed in their individual curve evolutions over time for each batch.

Preliminarily, the behavior of the ammonium polyphosphate (AP-IC) specimens can be evaluated (Figure 5a). All these samples showed a consistent temperature increase during the flame exposure. The IC specimen displayed a more rapid temperature rise compared to the AP-IC samples, indicating that the presence of an additional ammonium polyphosphate in the AP-IC coatings provided a beneficial thermal shielding effect on the standard intumescent coating’s (IC) insulating performance.

Notably, even after the flame was extinguished (the “fire-off” section on the right of the figure), the specimens continued to show a temperature increase before reaching their maximum, then gradually decreasing. The unexposed UV AP-IC-0 specimen exhibited a maximum temperature of approximately 20 °C lower than the reference IC specimen. As the UV exposure time increased, the thermal response of the AP-IC coatings slightly changed. Specifically, a reduction in the maximum temperature was observed, suggesting that this class of coatings offers a superior shielding role compared to the commercial reference. The best results were obtained for the AP-IC-600 specimen (after 600 h of UV exposure), which registered a maximum temperature of about 170 °C. Furthermore, all AP-IC specimens demonstrated their temperature peak during the fire-off phase at longer times than the IC specimen, confirming their superior active flame behavior.

Similar observations can be made for the specimens incorporating recycled glass as a filler (RG-IC, Figure 5b). Analyzing the performance of the unaged RG-IC-0 sample in more detail, it demonstrates insulating behavior comparable to the unaged AP-IC-0 at low flame exposure times. However, at longer exposure durations (beyond 40 s), a progressive deviation is noted, with the temperature increasing to values approaching that of the standard IC batch. Indeed, after 70 s, the RG-IC-0 sample registered a temperature of 198 °C, which is approximately 10 °C lower than the IC sample. Notably, UV aging at increasing exposure times consistently induced an appreciable decrease in temperature throughout the entire fire test, consequently reducing the maximum temperature reached. This beneficial effect of UV aging on thermal insulation becomes more pronounced with longer UV exposure durations, potentially suggesting that UV exposure enhances the long-term fire-retardant properties of these coatings.

Finally, Figure 5c provides a comparison of the backside temperature evolution for the series of cork-based intumescent coatings (CK-IC). The performance of the non-aged coating, CK-IC-0, demonstrates a significantly different temperature evolution compared to the commercial reference coating. This divergence strongly indicates the excellent barrier action provided by the cork filler, which substantially enhances the thermal insulation properties of the coating. Furthermore, even after extended UV exposure times, up to 900 h, no significant variations were detected in the temperature evolution trend over time. The curves for the aged samples remained quite similar to the unaged ones, both in their overall trend and the maximum temperature values achieved. This consistent performance underscores the good stability of the cork-filled intumescent coatings even after prolonged environmental aging.

**Figure 6:**
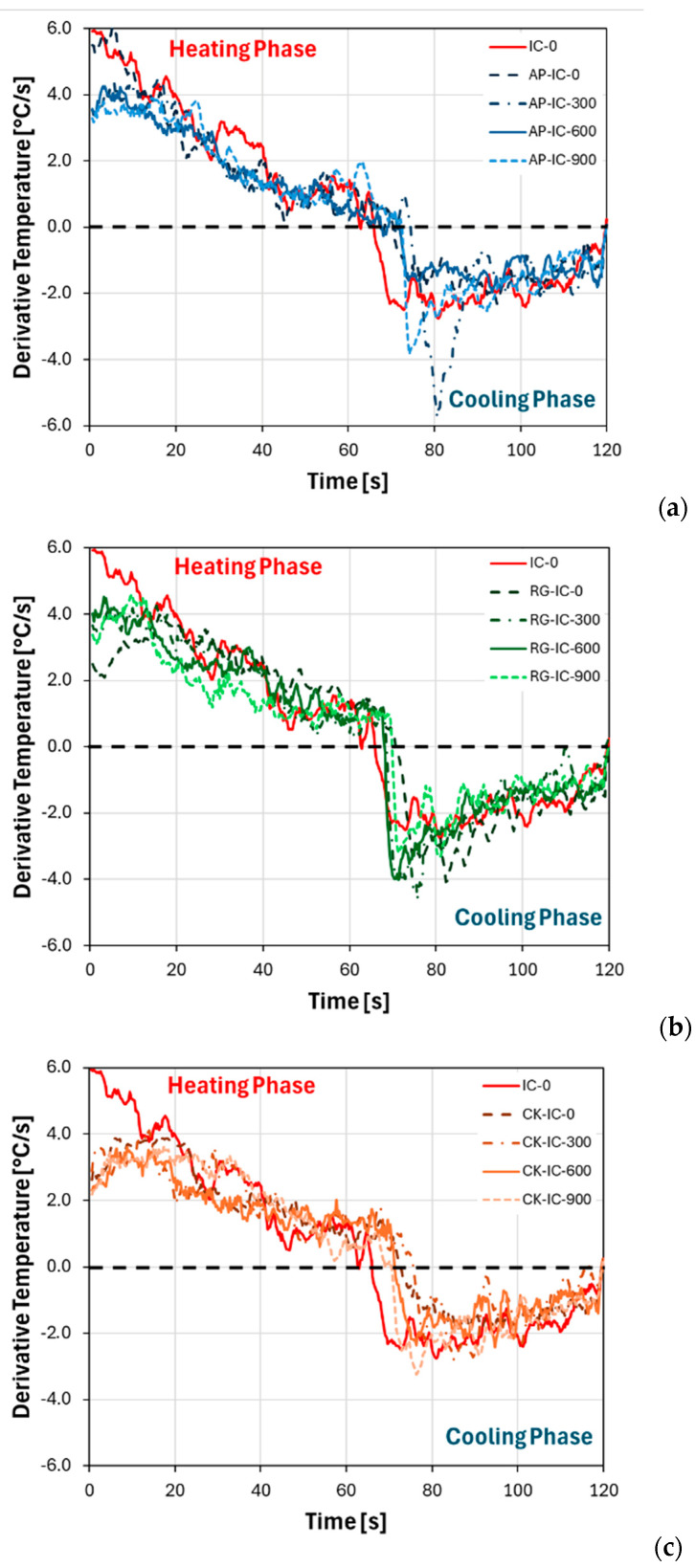
Derivative temperature evolution of (**a**) AP-IC, (**b**) RG-IC, and (**c**) CK-IC coatings at increasing UV aging time. IC coating as reference.

To further emphasize the distinctions in temperature evolution over time, it could be beneficial to analyze the heating and cooling rates of the curves. In this regard, Figure 6 presents the derivative temperature trend, which was calculated as the slope of the temperature-over-time curve, as illustrated in Figure 5. This approach provides a clearer understanding of how quickly the temperature changes throughout the test.

The derivative temperature of the uncoated steel substrate (SS), omitted from Figure 6 for graphical reasons but displayed in the Supplementary Materials Section for clarity, and the reference intumescent coating (IC), consistently registers higher values compared to all other functionalized coatings. This indicates a significantly faster temperature rise for these samples. Notably, the batch containing cork powder (CK-IC) exhibits the best thermal stability, characterized by generally low heating rate values throughout the entire fire test. Even during the initial phase of flame exposure, its heating rate remains remarkably below 4 °C/s (Figure 6c). Furthermore, Figure 6b illustrates that the recycled glass-functionalized intumescent coating (RG-IC) maintains a relatively effective derivative temperature curve at low exposure times, specifically around 4.5 °C/s. However, the AP-IC intumescent coating only shows a suboptimal derivative temperature trend for the untreated (unaged) sample, for which a higher initial heating rate is observed (Figure 6a). The observed decrease in heating rate across all coated samples, compared to the uncoated substrate, is not solely attributable to the stabilization of the flame temperature. More critically, it is also linked to the formation of a porous structure within the carbonaceous char layer generated during the intumescence process. This barrier action is further enhanced and accelerated by the release of hot gases trapped within this newly formed, expanding layer during the carbon foaming process, effectively pushing the char away from the heat source and improving insulation [30].

To comprehensively quantify and compare the fire resistance capabilities of the various coating formulations, a set of key performance indicators was derived from the fire exposure tests. Table 2 presents a summary of these critical parameters for all investigated coatings. These parameters include the maximum temperature reached on the backside of the substrate, the maximum heating rate observed during the test, and the crossover point, which is the specific time when the derivative temperature transitions from positive to negative, indicating a shift in thermal response from heating to cooling.

**Figure 7:**
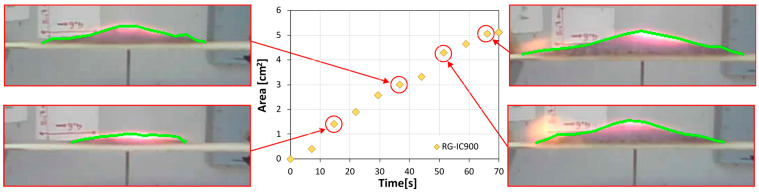
Evolution of the char layer’s surface area over time, accompanied by corresponding cross-sectional views, for the RG-IC-900 sample.

To better assess how the intumescence behavior of different coatings—each incorporating various sustainable fillers—evolved during the fire test, the swelling profiles of all coatings were recorded throughout the duration of the experiment. This was achieved through digital image analysis, employing a custom-built Python 3.8 script to quantify the geometrical shaping parameters of the porous char layer as it gradually foamed over time. As a reference example, Figure 7 presents the cross-sectional area results for the char layer generated by the glass-filled coating (RG-IC-900 sample), accompanied by its cross-section digital images recorded at various time intervals.

The cross-sectional area of the porous char layer expands over time when exposed to fire. Around 20 s of fire exposure, the curve plotting this area shows a distinct knee, related to the activation of the intumescence process. This activation leads to a rapid increase in the foamed char’s area due to swelling. Cross-sectional images of the sample at this time range corroborate this sudden growth in both the width and thickness of the intumescent layer. This expansion only slightly slows near 60 s of exposure, evidenced by a reduced slope in the curve. Further examination of cross-sectional images at longer exposure times confirms this observation, revealing a largely similar shape, which indicates the intumescent process for the RG-IC sample is nearly complete.

**Figure 8:**
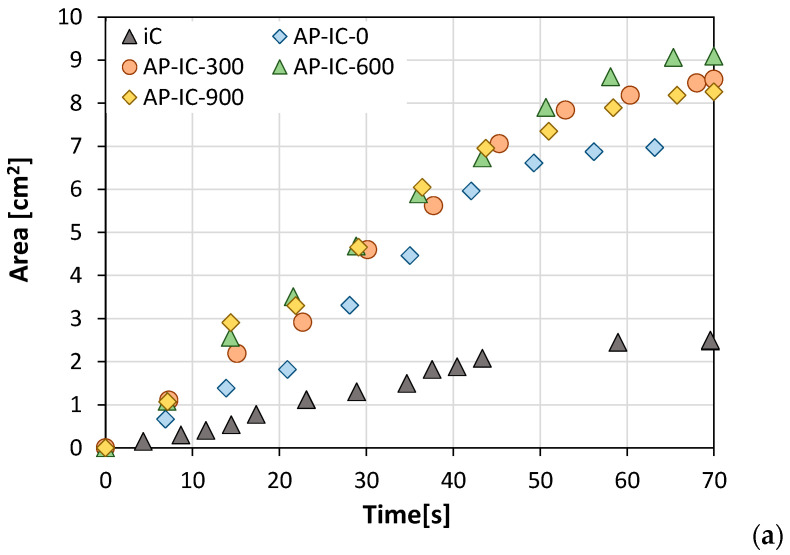

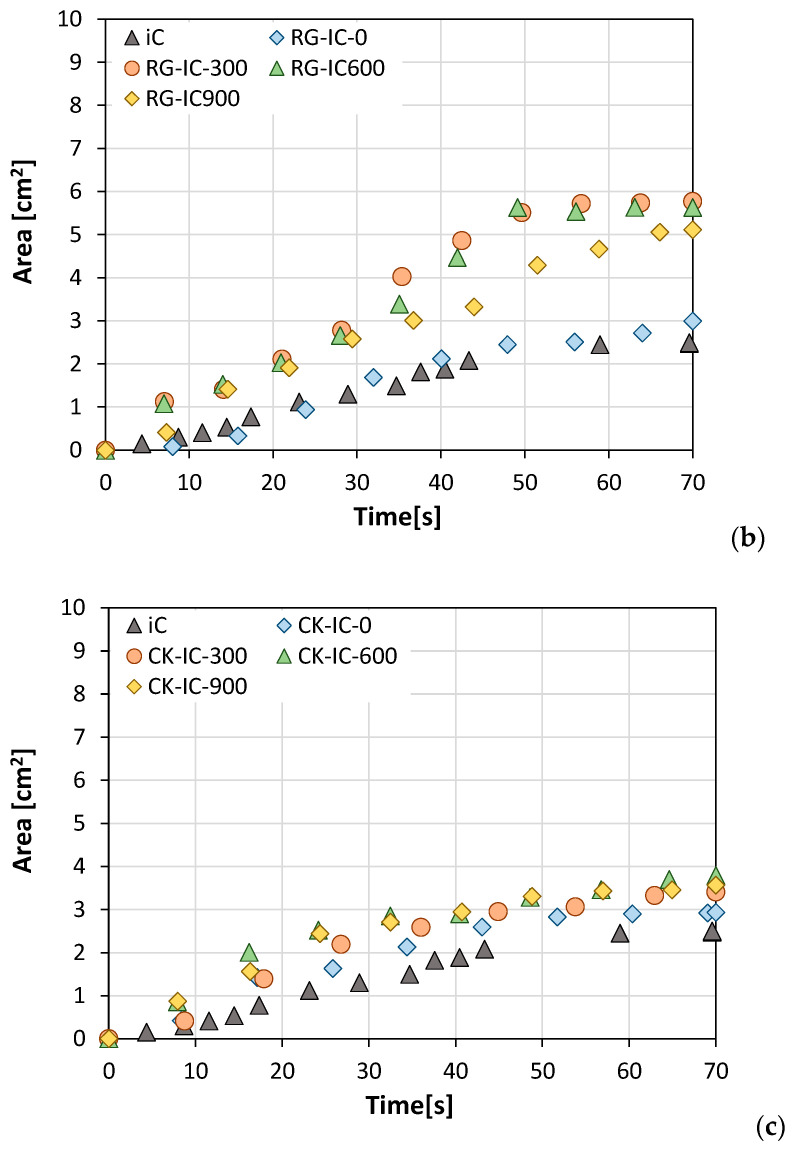
Cross-section intumescent area foamed during fire resistance test for (**a**) AP-IC, (**b**) RG-IC, and (**c**) CK-IC coatings at increasing UV aging times. IC coating as reference.

With the purpose of better understanding how UV aging impacts the crucial intumescent performance across different coating formulations, Figure 8 presents the cross-sectional intumescent area for all coated samples as a function of increasing UV aging time. Error bars were intentionally omitted from this plot to maintain optimal readability of the data points; however, it is important to note that the error achieved in the area measurements is approximately 20%. The slope of each curve directly correlates with the intumescence effect: a steeper upward slope signifies a greater and more effective expansion of the coating upon exposure to fire.

In the initial stages of the fire resistance test, all specimens, regardless of their specific formulation or UV exposure time, demonstrated roughly comparable intumescence properties, exhibiting similar linear trends in their expanding area curves. However, a distinct divergence in behavior became apparent at longer exposure times, as follows:

- The CK-IC (cork-filled) batch showed a rapid stabilization of its intumescent area, indicating the saturation of its foaming process. Notably, its performance experienced a slight increase due to UV aging exposure. For instance, the intumescent area for CK-IC-0 (unaged) was approximately 3.0 cm^2^, while CK-IC-900 (900 h UV aged) reached 3.7 cm^2^, representing a 23% increase. This unexpected enhancement in intumescence after UV aging for the cork-filled coating may be attributed to coupled sensitive photo-oxidation of the cork particles and the resin matrix [31]. This could lead to the formation of new functional groups [32] that serve as additional gas sources or charring agents when exposed to fire, thereby promoting a more robust or stable char structure [33]. Additionally, UV exposure may induce chemical modifications (e.g., cross-linking or oxidation) in the polymer matrix [34,35], which, upon heating, could enhance char formation and contribute to a more rigid and mechanically stable char.
- Conversely, the RG-IC (recycled glass-filled) batch also exhibited rapid stabilization but demonstrated a notably better foaming action, evidenced by an approximately 40% higher intumescence than the CK-IC batch. Unlike the cork-based samples, the recycled glass batch showed a significant dependence on UV aging exposure. Its intumescent behavior increased substantially, as confirmed by the foamed cross-sectional area that doubled in the UV-aged samples. However, no large differences were identified among the various UV-aged RG-IC samples. The increased intumescence with UV aging in the recycled glass-filled coatings could be ascribed to some synergistic factors. Coupled with resin modification as previously indicated, UV radiation might induce surface modifications on the glass particles, added as filler, potentially creating more reactive sites or altering their interaction with the polymer matrix and intumescent agents [36]. This could facilitate more efficient gas release or improve the mechanical integrity of the char, leading to enhanced expansion. The lack of significant difference between the UV-aged samples suggests that the primary UV-induced effect reaches saturation relatively early in the aging process.
- Meanwhile, the AP-IC (ammonium polyphosphate-filled) batch demonstrated an improved intumescence profile. Around 30–40 s, their area curves sharply increased due to the efficient foaming action supplied primarily by the ammonium polyphosphate (APP) within the formulation, significantly amplifying the intumescent action. After 70 s, the AP-IC samples achieved cross-sectional intumescent areas ranging from 7.0 to 9.1 cm^2^. These results confirm the superior performance of the APP-filled coating, exhibiting an intumescent area approximately two times larger than the traditional IC (unfilled) formulation. The pronounced increase in intumescence for the ammonium polyphosphate (APP)-filled coating is primarily attributed to APP’s role as a potent acid source and gas-generating agent within the intumescent system. Upon heating, APP decomposes to release phosphoric acid, which then promotes the charring of carbonific agents (like pentaerythritol) and generates non-flammable gases (e.g., ammonia). This coordinated action drives the rapid and extensive foaming of the coating, creating a highly effective insulating char layer that significantly amplifies the intumescent action.

**Figure 9:**
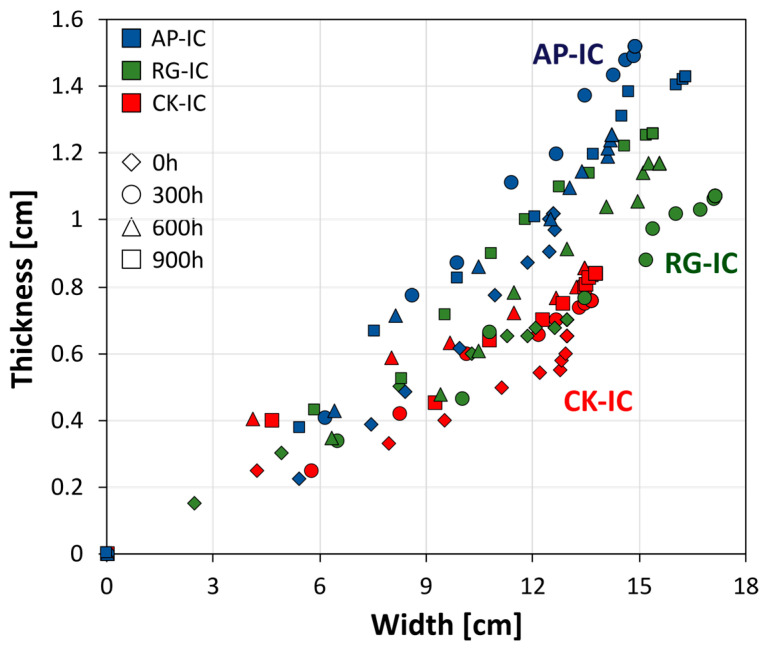
Width vs. thickness relationship for the cross-sectional intumescent area foamed during fire resistance tests for all batches: AP-IC (markers in blue),RG-IC (markers in green), and CK-IC (markers in red), at increasing UV aging times (diamond, circle, triangle, and square markers for 0 h, 300 h, 600 h, and 900 h, respectively).

With the purpose of providing further insights into how the shaping of the intumescent layer is influenced by the characteristics of each batch and the extent of UV degradation, Figure 9 illustrates the relationship between the width and thickness of the cross-sectional intumescent area foamed during fire resistance tests for the three different batches of intumescent coatings: AP-IC (blue markers), RG-IC (green markers), and CK-IC (red markers). Each batch’s performance was further differentiated by its exposure to increasing UV aging times, represented by distinct marker shapes: diamond for 0 h, circle for 300 h, triangle for 600 h, and square for 900 h.

The figure distinctly reveals three separate clusters, each representing a unique type of intumescent coating. The CK-IC family forms a well-defined cluster in the lower region of the graph, consistently displaying lower width and thickness values in its foamed intumescent layer. In contrast, the RG-IC family generally occupies a middle ground, characterized by higher average thicknesses and greater widths when compared to the CK-IC coatings. Notably, the AP-IC family stands out in the upper portion of the graph. While its intumescent width is comparable to that of the RG-IC coatings, the AP-IC family consistently achieves slightly superior average thicknesses.

This differentiation underscores that the AP-IC system exhibits the most favorable intumescence characteristics among the three tested batches. Its performance is characterized by a more expanded and consistent foamed region, as required for effective fire protection. This higher intumescence, identifiable in both significant area extension and enhanced coating thickness, makes the AP-IC system particularly well-suited for demanding naval applications. Importantly, the figure also indicates that the CK-IC and RG-IC batches—notably characterized by sustainable recycled glass and cork fillers, respectively—offered potentially interesting intumescent properties compared to the commercial reference, signifying a promising step towards more eco-friendly fire-resistant solutions.

The ability to form a thick, extensive insulative barrier is paramount for improving fire resistance on ships, where structural integrity and occupant safety during a fire are critical considerations, potentially allowing for extended evacuation times and improved containment of fire events.

Furthermore, the effective intumescent properties of recycled glass and cork fillers open new research avenues for novel hybrid composite coatings. Future activities will be aimed at synergistically combining these sustainable fillers with tailored amounts of AP systems, offering a compelling and potentially attractive solution for companies.

The investigation could be further refined by a more detailed analysis of the combustion processes during flame contact, such as fume analysis. Additionally, varying the oxygen content in the atmosphere would provide a broader understanding of the material’s behavior in several environments. This extended analysis, while rooted in naval sector applications, could also reveal the potential of these innovative intumescent materials for fire resistance in diverse other contexts.

This not only enhances fire resistance in various materials but also aligns with the growing demand for sustainable and eco-friendly solutions. As industries prioritize safety and ecological responsibility, these sustainable intumescent coatings, which effectively slow combustion and boost thermal protection, will become indispensable for developing safer, more resilient products across consumer and industrial applications, especially in critical sectors like naval engineering.

## 4. Conclusions

This study investigated the morphology, chemical composition, and fire resistance performance of various intumescent coatings, including the enhanced ammonium polyphosphate (AP-IC) formulation, and two novel formulations incorporating sustainable fillers: recycled glass (RG-IC) and cork (CK-IC), all tested at varying UV aging exposures.

Microscopic analysis revealed that most coatings exhibited a regular and dense texture with consistent particle distribution, indicative of successful material integration and good interfacial bonding, particularly evidenced by the absence of cracks. The commercial intumescent reference (IC sample), however, showed more inhomogeneities. Chemical analysis confirmed the primary composition of the coatings, highlighting a higher silicon content in the RG-IC sample due to the recycled glass addition. FTIR spectroscopy provided further insights into the chemical structure of the unaged coatings and demonstrated the effects of UV aging. Increasing UV exposure led to the formation of C=O groups and P-O-P linkages, indicating photo-oxidation of the resin and hydrolysis of ammonium polyphosphate, respectively, particularly in the CK-IC sample.

Fire resistance tests demonstrated the higher performance of the functionalized intumescent coatings compared to the commercial IC reference. All intumescent samples significantly delayed temperature rise and reduced maximum temperatures on the backside of the substrate. The AP-IC system reliably displayed the most favorable intumescence characteristics, with its AP-IC-600 sample achieving a low maximum temperature of 167.3 °C and a low maximum heating rate of 4.3 °C/s, outperforming the IC reference at 210.0 °C and 5.9 °C/s. This performance is attributed to the formation of a more expanded and consistent foamed region, making AP-IC particularly well-suited for demanding applications like naval engineering, where extensive insulative barriers are crucial for structural integrity and occupant safety.

Furthermore, the CK-IC and RG-IC batches, incorporating sustainable recycled glass and cork fillers, respectively, also exhibited promising intumescent properties when compared to the commercial reference. While the CK-IC batch showed a rapid stabilization of its intumescent area, its performance increased by 23% (from approximately 3.0 cm^2^ for CK-IC-0 to 3.7 cm^2^ for CK-IC-900) with UV exposure, suggesting beneficial photo-oxidation effects. The RG-IC batch demonstrated notably better foaming action than CK-IC, with its foamed cross-sectional area doubling for UV-aged samples compared to unaged ones and reaching a maximum temperature of 166.9 °C for RG-IC-900. The ability of these sustainable fillers to contribute to effective fire resistance opens exciting avenues for novel hybrid composite coatings. Combining these eco-friendly materials with tailored AP systems presents a compelling and attractive solution for industries seeking to enhance fire safety while simultaneously addressing the growing demand for sustainable materials. The continuous development of such sustainable intumescent coatings, capable of effectively slowing down combustion and bolstering thermal protective performance, will be indispensable in developing safer and more resilient products across a wide range of consumer and industrial applications, especially in critical sectors like naval engineering.

## Figures and Tables

**Table 1:** Codes and characteristics of all coatings.

Code	Characteristics
IC	Standard intumescent coating
AP-IC	Intumescent coating with increased ammonium polyphosphate
RG-IC	Intumescent coating with recycled glass microparticles
CK-IC	Intumescent coating with cork particulates

**Table 2:** Main summary parameters of the fire resistance performance for all investigated coatings.

Sample Code	Max Temperature [°C]	Max Heating Rate [°C/s]	Cross-Over Point [s]
SS	430.8 ± 37.9	9.9 ± 0.9	82.9 ± 7.4
IC	210.0 ± 19.1	5.9 ± 0.5	62.6 ± 6.0
AP-IC-0	187.0 ± 15.9	6.0 ± 0.5	67.0 ± 6.4
AP-IC-300	182.1 ± 15.1	4.5 ± 0.4	70.4 ± 6.5
AP-IC-600	167.3 ± 16.4	4.3 ± 0.4	67.3 ± 6.3
AP-IC-900	179.5 ± 18.0	3.9 ± 0.4	72.4 ± 7.2
RG-IC-0	198.7 ± 19.9	4.0 ± 0.4	70.4 ± 6.5
RG-IC-300	190.1 ± 18.1	4.3 ± 0.4	68.3 ± 5.9
RG-IC-600	188.9 ± 16.6	4.5 ± 0.5	67.8 ± 5.5
RG-IC-900	166.9 ± 14.0	4.6 ± 0.4	69.8 ± 6.2
CK-IC-0	188.7 ± 17.0	4.0 ± 0.4	72.3 ± 6.8
CK-IC-300	193.0 ± 16.6	3.9 ± 0.4	75.5 ± 7.0
CK-IC-600	183.9 ± 16.9	3.6 ± 0.4	71.1 ± 6.8
CK-IC-900	183.6 ± 17.1	3.6 ± 0.4	68.8 ± 6.7

## Data Availability

Data are contained within this article.

## Supplementary Materials

The following supporting information can be downloaded at: https://www.mdpi.com/article/10.3390/polym17121659/s1. Figure S1. Temperature profile (a) and Derivative temperature profile (b) at increasing time for SS sample. Figure S2. Investigated coatings after burning.
