# Supplementary material for: Durability Assessment of Eco-Friendly Intumescent Coatings Based on Cork and Waste Glass Fillers for Naval Fire Safety
Source: Polymers (Basel). 2025 Jun 15;17(12):1659. doi: 10.3390/polym17121659 (PMC12197251; doi:10.3390/polym17121659)
Supplement: Supplementary file 1 [file polymers-17-01659-s001.zip › polymers-3705060-supplementary.pdf]

Supplementary Material

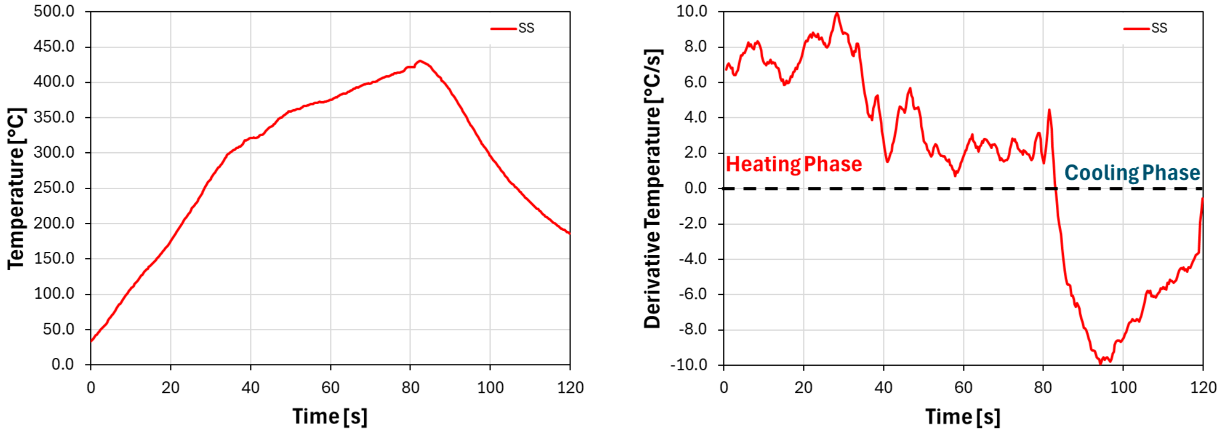

a)

Figure S1 Temperature profile a) and Derivative temperature profile b) at increasing time for SS sample

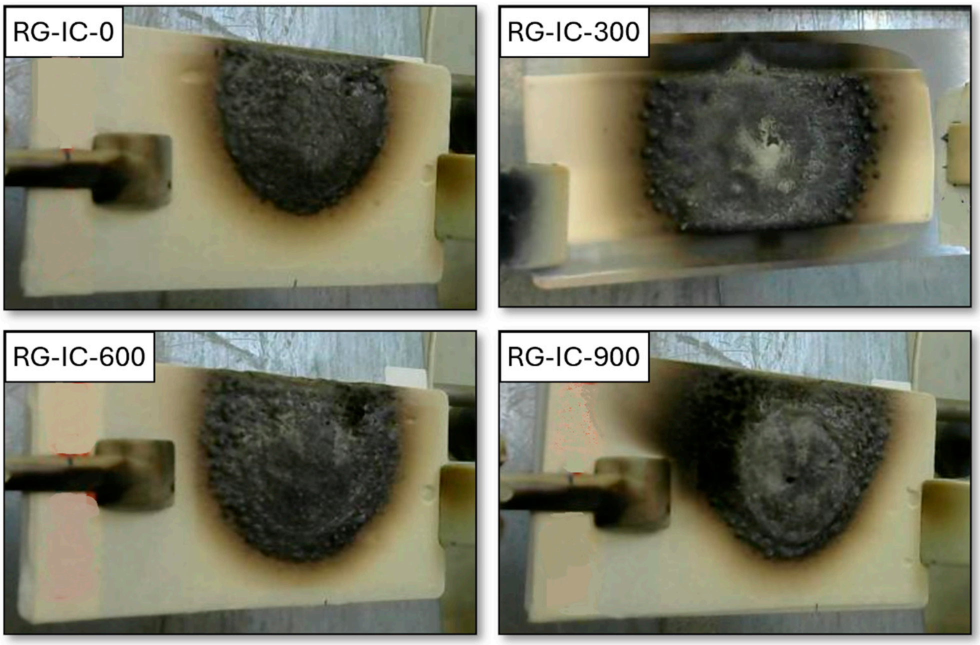

Figure S2 Investigated coatings after burning
